# The low excretor phenotype of glutaric acidemia type I is a source of false negative newborn screening results and challenging diagnoses

**DOI:** 10.1002/jmd2.12217

**Published:** 2021-04-05

**Authors:** Adam J. Guenzel, Patricia L. Hall, Anna I. Scott, Christina Lam, Irene J. Chang, Jenny Thies, Carlos R. Ferreira, Pavel Pichurin, William Laxen, Kimiyo Raymond, Dimitar K. Gavrilov, Devin Oglesbee, Piero Rinaldo, Dietrich Matern, Silvia Tortorelli

**Affiliations:** ^1^ Biochemical Genetics Laboratory, Mayo Clinic Rochester Minnesota USA; ^2^ Department of Human Genetics Emory University Atlanta Georgia USA; ^3^ Biochemical Genetics Laboratory Seattle Children's Hospital Seattle Washington USA; ^4^ Division of Genetic Medicine, Department of Pediatrics University of Washington and Seattle Children's Hospital Seattle Washington USA; ^5^ National Human Genome Research Institute (NHGRI) Bethesda Maryland USA; ^6^ Division of Clinical Genomics Mayo Clinic Rochester Minnesota USA

**Keywords:** glutaric acidemia, glutarylcarnitine, glutaryl‐CoA dehydrogenase, low excretor, newborn screening

## Abstract

**Background:**

Glutaric acidemia type I (GA1) is an organic acidemia that is often unrecognized in the newborn period until patients suffer an acute encephalopathic crisis, which can be mistaken for nonaccidental trauma. Presymptomatic identification of GA1 patients is possible by newborn screening (NBS). However, the biochemical “low‐excretor” (LE) phenotype with nearly normal levels of disease metabolites can be overlooked, which may result in untreated disease and irreversible neurological sequelae. The LE phenotype is also a potential source of false negative (FN) NBS results that merits further investigation.

**Methods:**

Samples from six LE GA1 patients were analyzed by biochemical and molecular methods and newborn screen outcomes were retrospectively investigated.

**Results:**

Five LE GA1 patients were identified that had normal NBS results and three of these presented clinically with GA1 symptoms. One additional symptomatic patient was identified who did not undergo screening. Semiquantitative urine organic acid analysis was consistent with a GA1 diagnosis in two (33%) of the six patients, while plasma glutarylcarnitine was elevated in four (67%) of the six and urine glutarylcarnitine was elevated in four (80%) of five patients. Five *GCDH* variants were identified in these patients; three of which have not been previously linked to the biochemical LE phenotype.

**Conclusions:**

The data presented here raise awareness of potential FN NBS results for LE GA1 patients. The LE phenotype is not protective against adverse clinical outcomes, and the possibility of FN NBS results calls for high vigilance amongst clinicians, even in the setting of a normal NBS result.

**Synopsis**

Patients with the low excretor biochemical phenotype of glutaric acidemia type I are an ongoing source of potential false‐negative laboratory results, but are subject to severe neurological sequelae if untreated.

**Abbreviations**
3OHGA: 3‐hydroxyglutaric acid
C5DC: glutarylcarnitine
FIA‐MS/MS: flow injection analysis‐tandem mass spectrometry
FN: false negative
GA: glutaric acid
GA1: glutaric acidemia type I
GCDH: glutaryl‐CoA dehydrogenase
GC‐MS: gas chromatography‐mass spectrometry
HE: high excretor
LE: low excretor
NBS: newborn screening
RUSP: recommended uniform screening panel

## 1 INTRODUCTION

Glutaric acidemia type I (GA1, OMIM 231670) is an autosomal recessive neurometabolic disorder caused by biallelic pathogenic variants in *GCDH* resulting in deficiency of glutaryl‐CoA dehydrogenase (GCDH). GCDH converts glutaryl‐CoA to crotonyl‐CoA within the mitochondria. GCDH deficiency leads to accumulation of glutaryl‐CoA, which is then converted into glutaric acid (GA) and 3‐hydroxyglutaric acid (3OHGA) or excreted as glutarylcarnitine (C5DC) after esterification with carnitine, causing secondary carnitine deficiency.

The clinical presentation of GA1 includes macrocephaly that is often evident at birth and throughout infancy. Postnatal development generally progresses normally and patients may go undetected until suffering an acute encephalopathic crisis. This crisis is often precipitated by febrile illness with concurrent emesis and dehydration during the first 6 years of life and result in striatal injury, gliosis, and neuronal loss and lead to dystonia and choreoathetosis.^1^ Imaging studies have revealed widened Sylvian fissures and arachnoid cysts.^2^ Subdural and retinal hemorrhages may be mistaken for nonaccidental trauma when the diagnosis is unknown.^3^ Treatment with a low‐lysine diet, l‐carnitine supplementation, and prompt initiation of emergency protocols during times of illness is necessary for optimal clinical outcome.^1^, ^4^ Therefore, clinical manifestations in GA1 are strongly dependent upon adherence to dietary therapy and rapid treatment during acute illnesses.^5^ GA1 is included in the recommended uniform screening panel (RUSP) in the United States and other countries with newborn screening (NBS) programs, and is identified by detecting elevated levels of C5DC in dried blood spot cards using flow injection analysis‐tandem mass spectrometry (FIA‐MS/MS).^6^ It is recommended that newborns with a positive screen undergo repeat analysis of the same DBS along with additional analysis of disease metabolites in blood or urine.^1^ Early detection has had a positive effect on the neurological outcomes of many patients that have received adequate presymptomatic treatment by preventing irreversible neurologic damage and encephalopathic crises.^7^

Two distinct biochemical phenotypes exist amongst GA1 patients. Some patients are classified as high excretors (HEs), characterized by large amounts of GA and 3OHGA in urine, whereas others are low excretors (LEs) with modest (GA <100 mmol/mol creatinine) or normal urinary levels.^8^ Previous studies have successfully identified genotypes that more commonly result in either high or low levels of these biochemical analytes,^9^, ^10^, ^11^ but no correlation has been observed with clinical phenotype as both groups can suffer the most severe neurological manifestations of GA1.^9^ Further investigation of GA1 LEs is important because they are a potential source of false negative (FN) NBS results and can be missed by urine organic acid (OA) and plasma acylcarnitine testing, the traditional diagnostic methods for GA1.^12^

Urinary excretion of C5DC is a specific biochemical marker that is elevated in both LE and HE GA1 patients.^13^ Here we present data from several cases that highlight the difficulty in identifying LE patients, both by NBS and standard biochemical testing, and reinforce the utility of targeted urine C5DC analysis for complete GA1 diagnostic evaluation.

## 2 MATERIALS AND METHODS

### 2.1 Patients

A total of six LE GA1 patients are included in this study. LE GA1 was defined as a patient with a molecular and/or clinical diagnosis of GA1 including biochemical data with a urine glutaric acid excretion of <100 mmol/mol creatinine as defined previously.^8^ A single referral laboratory identified a small cohort (n = 2) of LE GA1 cases that were identified as a result of symptomatic investigation and found to have a normal NBS result. Communication with collaborators at other institutions identified additional LE patients with similar laboratory findings (n = 4). Molecular analysis of *GCDH* was available for five (83%) patients. NBS findings and clinical presentations are summarized (Table 1). IRB approval was acquired at all institutions either with a local IRB or by acceptance of the protocol established at Mayo Clinic under IRB 15‐005393 as the lead organization for this study.

**TABLE 1:** Characteristics of GA1‐low excretor patients

Case	Age at Dx	Variant 1	Variant 2	NBS method	Newborn screening					Urine C5DC			Urine organic acid		Plasma C2 (μmol/L)	Plasma C5DC (μmol/L)	Clinical presentation
					Result	AOC	Result	AOC rpt	Result rpt	AOC	(μmol/mmol creat)	C5DC/tAC	Glutaric (μmol/mmol creat)	3OHGA (qualitative)			
Laboratory control values							<0.13		<0.13		<1.5	<0.05	<14	Trace	Age dependent	<0.1	
1	8 mo.	p.Met405Val	p.Ala304Gly	Underivatized	Normal	—	—	—	—	3 mo.	**7.8**	**0.76**	<14	**Elevated**	N/A	**0.35**	Seizures and white matter abnormalities
2	1 y	N/A	N/A	Underivatized	Normal	—	—	—	—	N/A	N/A	N/A	3	**Elevated**	**21.6** (normal <17.83)	**0.43** ^a^	Dystonia, global developmental delay
3	3 y	p.Arg402Trp	p.Ala421Thr	Derivatized	Normal	1 h	**0.13**	16 d	0.04	3 y	1.1^a^	0.1^a^	1	Trace	5.2	0.1	Asymptomatic
4	18 mo.	p.Arg402Trp	p.Ala421Thr	Derivatized	Normal	24 h	**0.16**	6 d	0.08	24 mo.	**1.6** ^a^	0.1^a^	3	Trace	13.2	**0.14**	Acute motor regression
5	New born	p.Arg402Trp	p.Ala421Thr	Derivatized	Normal	24 h	0.16	No repeat		4 mo.	**2.3** ^a^	0.1^a^	2	Trace	9.6	**0.15**	Asymptomatic
6	10 mo.	p.Ile250Thr	p.Ile250Thr	Not performed		—	—	—	—	8 y	**1.7**	**0.14**	Not detected	Trace	N/A	<0.1	Acute motor/developmental regression

*Note:* C5DC/tAC = C5DC/sum of (C0, C2, C3, C4, C5, C6, C7, C8, C10, C12, C14, C16, and C18). Elevated laboratory values indicated with bold text.

Abbreviations: AOC, age of collection; GA1, glutaric acidemia type I.

^a^ Known carnitine supplementation at time of sample collection.

### 2.2 Quantitative procedures

Urine organic acid and plasma acylcarnitine analyses were performed at CLIA‐certified clinical biochemical genetics laboratories in the United States utilizing standard methods of gas‐chromatography‐mass spectrometry (GC‐MS) and FIA‐MS/MS, respectively. Urine acylcarnitine analysis was performed as described previously by Tortorelli et al.^13^ Glutarylcarnitine was identified by identification of a precursor ion MW = 388 m/z product ion MW = 85 m/z and recorded as both a value normalized to mmol creatinine and a ratio of glutarylcarnitine to total acylcarnitine species detected by this testing methodology (C0, C2, C3, C4, C5, C6, C7, C8, C10, C12, C14, C16, and C18). C5DC reference ranges were set above the maximum value observed in a cohort of 382 samples obtained from leftover, anonymized specimens submitted to the laboratory for analysis and below any previously observed GA1‐positive patient values.

## 3 RESULTS

### 3.1 Patients

Clinical, biochemical, and genotype data from six patients with GA1 are summarized in Table 1. Patients 1 to 5 were born in the United States and received NBS testing according to local protocols in place at the time of their birth. Patient 1 presented clinically at 8 months of age with seizures and white matter changes on MRI. Biochemical testing including urine organic acids, plasma acylcarnitines, and urine C5DC were consistent with GA1 and two variants were identified in the *GCDH* gene. Patient 2 presented clinically at 1 year of age with global developmental delay, dystonia, and macrocephaly. Biochemical testing including urine organic acids and plasma acylcarnitines was consistent with GA1 and molecular analysis was not pursued. Patient 4 presented clinically at 12 months of age, primarily with acute motor regression and hypotonia. Molecular analysis of the *GCDH* gene revealed two variants. Initial biochemical testing of plasma acylcarnitines revealed a mild elevation of C5DC (0.14 μmol/L, controls <0.1 μmol/L) with minimal abnormalities detectable in urine organic acids. Urine C5DC analysis performed after initiation of carnitine supplementation was also mildly elevated. Patient 3 is an older sibling and patient 5 is a younger sibling to patient 4; both were diagnosed by molecular and biochemical methods after symptomatic presentation of patient 4. All three siblings had C5DC in their initial NBS (obtained within the first day of life) that was above the laboratory cutoff (0.13), but repeat NBS at 6 days (patient 4) and 16 days (patient 3) was normal and no additional testing was initially pursued. Patient 5 had initial NBS C5DC levels consistent with those seen in siblings, but the state lab had previously raised the C5DC cutoff (from 0.13 to 0.21) so this patient was reported as normal, but molecular sequencing was initiated because of family history. Patient 6 was born outside of the United States and presented clinically at 10 months of age with acute motor and developmental regression following an episode of gastroenteritis. They remained undiagnosed until the age of eight after seeking clinical care in the United States.

### 3.2 Biochemical findings

Semiquantitative organic acid analysis was performed on all patients and analysis of urine glutarylcarnitine was performed on five (83%) of the six patients (Table 1). Concurrent treatment including l‐carnitine supplementation was noted at the time of sample collection when applicable. Overall, urine C5DC was elevated (reference range: <1.5 μmol/mmol creatinine) in four (80%) of the five samples tested (median = 1.7, range: 1.1‐7.8).

### 3.3 Genotype analysis

**FIGURE 1:**
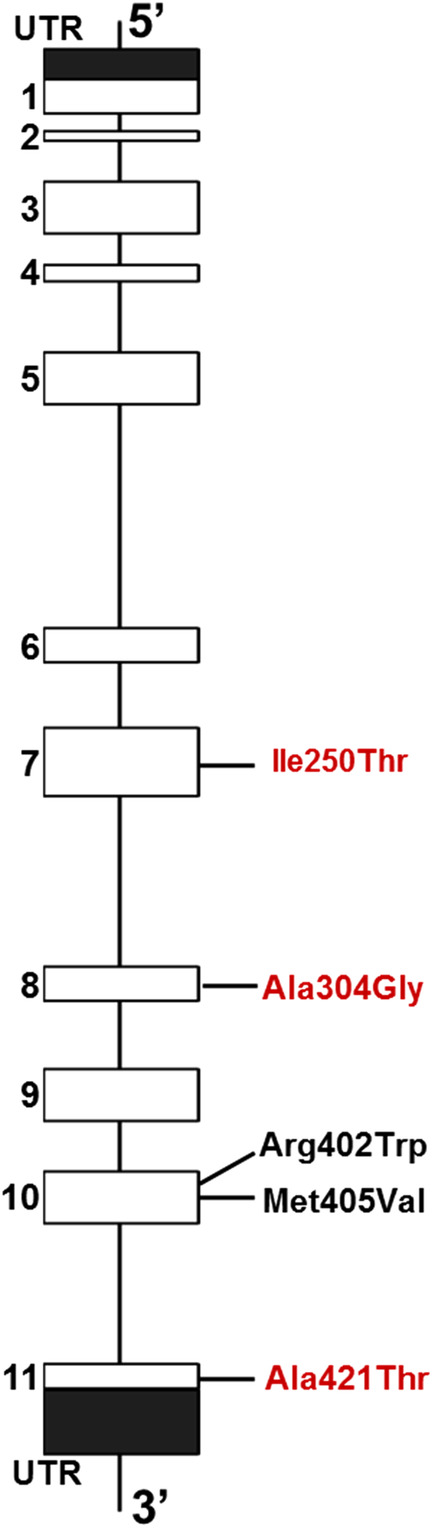
Diagram of human *GCDH* with variants identified in this study. Red text indicates novel variants that have not been previously associated with the LE phenotype. Black text indicates variants identified in this study that have previously been associated with the LE phenotype. LE, low‐excretor

Reporting and follow‐up (eg, parental studies) of molecular results varied by location of clinical care. Sequencing results were available for five (83%) patients and revealed five different variants in the *GCDH* gene (Figure 1). Two variants were identified in all five of the cases where sequencing was available (Table 1). Two (40%) of the five variants were previously reported with biochemical evidence correlating with the GA1 LE phenotype (Table 2).^9^, ^10^, ^11^, ^12^ Two *GCDH* variants (c.749T>C, p.Ile250Thr and c.911C>G, p.Ala304Gly) in our present study have been reported in GA1 patients, but have not, to our knowledge, been previously reported with sufficient biochemical data to correlate with the LE phenotype (Figure S2). One variant (c.1261G>A, p.Ala421Thr) was not previously reported in any GA1 patients.

**TABLE 2:** Characteristics of *GCDH* variants observed in patient cohort

Variant		Previously reported in GA1 patient	Previously reported in LE patient	# Alleles in patient cohort	ClinVar Interp	Gnomad			Proposed interpretation
p.	c.					# of Alleles	Allele frequency	# Homozygotes	
Ile250Thr	749T>C	Yes	No	2	None	0	N/A	0	Likely pathogenic
Ala304Gly	911C>G	Yes	No	1	VUS	0	N/A	0	Likely pathogenic
Arg402Trp	1204C>T	No	8, 9, 10	3	P/LP	89	3.15E−04	0	Pathogenic
Met405Val	1213A>G	No	10, 11	1	P	21	7.43E−05	0	Pathogenic
Ala421Thr	1261G>A	No	No	3	VUS	62	2.19E−04	0	Likely pathogenic

Abbreviations: GA1, glutaric acidemia type I; LE, low excretor.

## 4 DISCUSSION

This study examines six cases of GA1 with the LE phenotype and highlights diagnostic difficulties related to NBS and follow‐up investigation. The diagnosis of GA1 by urine organic acid analysis relies on identification of increased excretion of GA in the presence of 3OHGA, or the presence of 3OHGA alone for the LE phenotype. In these six patients, GA excretion was within normal limits in all cases and 3OHGA was definitively elevated by qualitative analysis in only two cases. Urine glutarylcarnitine quantitation was informative in four (80%) of the five cases where it was tested. These findings reinforce the value of urine glutarylcarnitine analysis and continued clinical vigilance for the diagnosis of GA1. Quantitation of C5DC in plasma is also a sensitive marker for GA1 (elevated in four (67%) of six patients); however, analysis of plasma acylcarnitines in the clinical lab regularly reveals mild elevations of C5DC, making this a less specific marker for GA1.^14^

GA1 is included in the RUSP for NBS in the United States, but biochemical heterogeneity has complicated detection of GA1 in screening and diagnostic laboratories. It is important to note that the LE phenotype represents a significant proportion (30%‐40%) of GA1 patients and they are equally likely to manifest severe complications of GA1.^9^, ^13^, ^15^ NBS for GA1 is based on quantification of C5DC in dried blood spots as part of the routine assay performed on almost all infants born in the United States. Acylcarnitine quantification methods using either a derivatized or underivatized analyses vary by state. Derivatization enables the formation of isobaric butyl esters of 3‐hydroxydecanoylcarnitine (C10OH) that can result in a higher concentration of C5DC in control samples. This leads to higher cutoffs to reduce the number of false positive screens. Due to the inherent pitfalls in GA1 screening, it is now recommended that all infants with positive initial NBS results undergo follow‐up screening utilizing a urine specimen in addition to, or in place of a repeat DBS analysis. In this cohort of five patients that were recently missed by NBS, two of these were screened by the underivatized method, and three patients from the same family were missed by the derivatized method. The data here are insufficient to draw any conclusions regarding the optimal screening method.

This study also identified two novel (c.749T>C, p.Ile250Thr and c.911C>G, p.Ala304Gly) variants in *GCDH* which had not previously been reported in individuals affected with GA1 (Table 2) and an additional variant (c.1261G>A, p.Ala421Thr) identified previously in a GA1 patient, but without evidence linking the variant to the LE phenotype.^16^ Of the novel variants identified in this study, all are classified as variants of uncertain significance according to current ACMG guidelines.^17^ With this data, we have expanded the list of variants that can be observed in the LE phenotype (Figure 1). However, some variants are associated with both HE and LE phenotypes. For example, the variant p.Arg402Trp has been reported in heterozygous LE GA1 patients *in trans* with p.Val400Met and p.Met405Val, but has also been reported in homozygous HE GA1 patients and compound heterozygous HE GA1 patients with p.Ala293Thr, p.Arg128*, c.10‐2A>G, and c.1209delGly.^9^, ^10^, ^11^ It is likely that the reduction in GCDH activity is a spectrum, and the combined impacts of both variants determine the overall biochemical phenotype; however, this has not been proven. The high number of uncertain variants is not unusual in NBS conditions with biochemical phenotypes. The combination of biochemical test results or clinical findings with suggestive or uncertain molecular findings is often not followed with additional testing to classify the variants more conclusively, such as copy number analysis or parental testing.^18^

The cases presented here highlight several unfortunate events where patients were not detected by NBS and went on to develop the neurological sequela associated with GA1. Presymptomatic treatment could have been initiated if these patients were detected during the asymptomatic newborn period. There are several options for improving NBS performance, including a tiered testing approach quantitating GA and 3OHGA in DBS.^19^ Tiered NBS for certain conditions allows an initial cutoff to be set low enough to detect all possible cases, while the higher specificity of a second tier test will limit the potential false positive results. Postanalytical tools such as Collaborative Laboratory Integrated Reports (CLIR; https://clir.mayo.edu) can improve performance by considering a larger profile and additional ratios, while adjusting for multiple covariates such as age and birth weight. An earlier version of postanalytical tools demonstrated significant performance improvement in NBS.^20^ Sequence analysis may provide clarity for GA1 screening results, but it does not offer conclusive evidence in every case. The combination of biochemical, molecular, and clinical findings needs to be carefully considered in each case. A high degree of awareness by clinicians is also important in the presence of suggestive clinical findings and a normal NBS result. Patients born with glutaric acidemia type I are often asymptomatic until they suffer an acute encephalopathic crisis, but early detection by NBS allows implementation of dietary restriction and rapid response to potential periods of metabolic decompensation.

## 5 CONCLUSIONS

NBS for GA1 and other disorders is an extremely valuable public health effort aimed at early diagnosis of treatable disorders. However, this study highlights the lack of sensitivity that still exists in screening for GA1 as evidenced by five FN NBS results of LE GA1 patients. Regardless of the biochemical phenotype, patients who receive early diagnosis and treatment through NBS have improved clinical outcomes compared to those identified symptomatically, supporting its inclusion in the US RUSP and state‐dependent expanded NBS panels. However, the confirmed FN results by NBS reinforce the need for clinician vigilance when clinical findings are suggestive of GA1 despite a normal NBS report. The LE phenotype provides particular diagnostic challenges in the NBS setting and in clinical laboratories using traditional testing methods, and these patients emphasize the importance of urine glutarylcarnitine analysis for the diagnosis of LE GA1 patients. As demonstrated in our present study, integration of biochemical data, obtained by specific analytical methods such as urine glutarylcarnitine analysis, clinical findings and molecular testing is imperative to accurately diagnose patients with GA1 in order to initiate early treatment and ensure their optimal clinical outcome.

## CONFLICT OF INTEREST

The authors declare no potential conflict of interest.

## ETHICS STATEMENT

All procedures followed were in accordance with the ethical standards of the responsible committee on human experimentation (institutional and national) and with the Helsinki Declaration of 1975, as revised in 2000 (5).

## PATIENT CONSENT STATEMENT

Informed consent was obtained from all patients for being included in the study. This article does not contain any studies with human or animal subjects performed by the any of the authors.
